# Geospatial Modeling of Deep Neural Visual Features for Predicting Obesity Prevalence in Missouri: Quantitative Study

**DOI:** 10.2196/64362

**Published:** 2024-12-17

**Authors:** Butros M Dahu, Solaiman Khan, Imad Eddine Toubal, Mariam Alshehri, Carlos I Martinez-Villar, Olabode B Ogundele, Lincoln R Sheets, Grant J Scott

**Affiliations:** 1 University of Missouri Institute for Data Science and Informatics Columbia, MO United States

**Keywords:** geospatial modeling, deep convolutional neural network, DCNN, Residual Network-50, ResNet-50, satellite imagery, Moran I, local indicators of spatial association, LISA, spatial lag model, obesity rate, artificial intelligence, AI

## Abstract

**Background:**

The global obesity epidemic demands innovative approaches to understand its complex environmental and social determinants. Spatial technologies, such as geographic information systems, remote sensing, and spatial machine learning, offer new insights into this health issue. This study uses deep learning and spatial modeling to predict obesity rates for census tracts in Missouri.

**Objective:**

This study aims to develop a scalable method for predicting obesity prevalence using deep convolutional neural networks applied to satellite imagery and geospatial analysis, focusing on 1052 census tracts in Missouri.

**Methods:**

Our analysis followed 3 steps. First, Sentinel-2 satellite images were processed using the Residual Network-50 model to extract environmental features from 63,592 image chips (224×224 pixels). Second, these features were merged with obesity rate data from the Centers for Disease Control and Prevention for Missouri census tracts. Third, a spatial lag model was used to predict obesity rates and analyze the association between deep neural visual features and obesity prevalence. Spatial autocorrelation was used to identify clusters of obesity rates.

**Results:**

Substantial spatial clustering of obesity rates was found across Missouri, with a Moran I value of 0.68, indicating similar obesity rates among neighboring census tracts. The spatial lag model demonstrated strong predictive performance, with an *R*^2^ of 0.93 and a spatial pseudo *R*^2^ of 0.92, explaining 93% of the variation in obesity rates. Local indicators from a spatial association analysis revealed regions with distinct high and low clusters of obesity, which were visualized through choropleth maps.

**Conclusions:**

This study highlights the effectiveness of integrating deep convolutional neural networks and spatial modeling to predict obesity prevalence based on environmental features from satellite imagery. The model’s high accuracy and ability to capture spatial patterns offer valuable insights for public health interventions. Future work should expand the geographical scope and include socioeconomic data to further refine the model for broader applications in obesity research.

## Introduction

### Overview

The prevalence of obesity has escalated to alarming levels globally, prompting an urgent need for innovative approaches to understand and combat this complex health issue. Spatial technologies, such as geographic information systems (GISs), remote sensing (RS), spatial machine learning (ML), and spatial analysis, have emerged as powerful tools in obesity research, offering novel insights into the environmental and social determinants of this epidemic. This paper delves into the current applications of spatial technologies in obesity research, highlighting how these tools can be used to unravel the intricate relationship between the built environment and obesity rates.

Furthermore, this paper explores the future promise of these technologies in advancing our understanding of obesity, guiding public health interventions, and shaping policies to create healthier and more equitable communities. Through a comprehensive examination of recent studies and advancements, this paper underscores the pivotal role of spatial technologies in transforming obesity research and ultimately contributing to the global fight against this pressing public health challenge.

### Objectives

This research aims to use deep convolutional neural networks (DCNNs) to examine medium-resolution satellite imagery, with the goal of predicting obesity rates using deep neural visual features (DNVFs). Concentrating on the 1052 census tracts in Missouri, the project seeks to provide a scalable method for predicting obesity prevalence using DNVFs. This could improve the accuracy of public health initiatives and policy decisions.

### Background

GISs, GPS, RS, and DCNNs such as Residual Network-50 (ResNet-50) are reshaping obesity research. GISs have been pivotal in depicting spatial distributions of obesity and its determinants, crafting metrics of obesity-promoting environments, and unveiling spatial patterns of obesity prevalence and obesogenic environmental attributes. GPS has predominantly been used to map individual movement patterns and, in conjunction with other instruments, to track behaviors contributing to obesity. The contribution of RS in supplying data on natural and built environments has been undervalued [1,2].

The integration of DCNNs, particularly ResNet-50, and the extraction of DNVFs have enhanced the precision of obesity predictions by analyzing satellite imagery and identifying subtle environmental features linked to obesity. When combined with spatial ML techniques, these advanced technologies are proving invaluable in providing more granular insights into obesogenic environments and individual exposure levels, thereby enriching our understanding of obesity’s etiology and the impact of various interventions on obesity rates in Missouri [1,3,4].

By 2030, it is projected that 38% of the global population will be overweight, while another 20% will be obese [5,6]. This alarming trend toward a worldwide epidemic of “globesity” has raised concerns about the rapid and significant shift in global health patterns. The United States, and specifically the state of Missouri, is no exception to this trend, with an increasing burden of individuals who are overweight and obese.

Changes in dietary patterns, particularly the consumption of energy-dense, nutrient-poor foods, are closely linked to the rising prevalence of overweight and obesity and their associated health complications. Therefore, this study aims to explore the spatial patterns of overweight and obesity among adults in Missouri and predict the obesity rate for each census tract to increase public health awareness [7].

### Significance

Earlier studies have shown links between different elements of the built environment and their effects on obesity and physical activity across various life stages [8-10]. Previous research has demonstrated a connection between obesity and various environmental factors, including the walkability of a region, land use patterns, urban sprawl, residential types, access to amenities such as recreational facilities and food outlets, socioeconomic deprivation, and perceived safety levels in a community [11-13]. In addition, proximity to and availability of natural spaces and sidewalks are linked to increased and more regular levels of physical activity, especially in urban settings [14,15].

While a relationship between obesity and the built environment is acknowledged, inconsistencies exist in the results of various studies and across different geographic regions concerning how specific features of the built environment influence obesity rates [11,16,17]. These discrepancies could arise from variations in the measurement methods and tools used in these studies, making it challenging to assess and compare the outcomes [18-20]. Furthermore, the measurement of these environmental features often requires substantial time and expenses and is susceptible to human error and bias [21,22]. There is a need for methodologies that provide standardized measurement criteria to enable comparisons between various studies [19,23,24]. Accurately assessing the impact of the built environment on obesity is crucial for designing and implementing successful community-based prevention and intervention programs [21,25,26].

In this paper, we introduce an innovative approach to extensively explore the relationship between adult obesity prevalence and different features of the spatial characteristics. This method uses a deep learning (DL) technique, using a DCNN to examine the physical characteristics of neighborhoods through medium-resolution (10 m) satellite imagery. Building on the foundational work of Maharana and Nsoesie [27] and Nguyen et al [28], who used DCNNs to analyze urban Google Street View (Google LLC) images focusing on predefined features, such as crosswalks, building types, and greenery, our study expands the scope to both urban and rural areas. Unlike Maharana and Nsoesie [27], who used a Visual Geometry Group-8 architecture and extracted features after training, we used the more advanced ResNet-50 architecture, using features extracted from a pretrained model to enhance efficiency and generalizability. Our approach provides a more comprehensive analysis of the DNVFs, identifying specific correlations with obesity rates at the census tract level across 1052 census tracts in Missouri. Moreover, our method is scalable, uses publicly available data and computing resources, allows for comparisons between various studies, and can be adapted to different geographic locations and regions.

### RS Applications in Obesity Research

RS data, typically stored as raster data, offer a highly efficient means of spatially representing obesity risk. Raster data consist of regular square grids, with each grid cell aggregating information over a corresponding area on Earth’s surface, such as high-resolution aerial photos and satellite images. These data can be generated from radiation recorded by sensors or through GIS-based interpolation techniques from discrete data collected at observation stations [29-32].

RS technology, which acquires information through spaceborne satellites or airborne sensors without direct physical contact, records the intensity of radiation reflected or emitted by objects and converts it into various land surface (eg, vegetation) and meteorological (eg, temperature) properties for user analysis.

This technology has been instrumental in diverse applications, including environmental monitoring and climate-sensitive disease risk modeling, by providing extensive environmental data [1,29,33-35].

In obesity research, RS plays a pivotal role by offering detailed environmental data that can be used to identify obesogenic factors, such as the availability of green spaces or urban walkability. This spatial information, when combined with other data sources, can help researchers understand the complex interplay between the environment and obesity risk. Vector data, which include points, lines, and polygons with geographical coordinates, complement raster data by providing precise location information for features relevant to obesity studies [36-39].

### Literature Review and Related Work

#### DL Applications in Urban and Environmental Health Research

DL techniques have become integral to understanding the complex dynamics between urban environments and public health outcomes. Through the application of convolutional neural networks (CNNs) and semantic segmentation architectures, these methodologies enable detailed analysis of spatial data for health-related urban studies.

#### CNNs in Urban Health Analysis

Pala et al [40] used transfer learning with a pretrained CNN to classify urban structures in high-resolution satellite imagery. This method facilitates the extraction of latent features indicative of various urban elements, critical for analyzing the impact of the urban environment on public health. The subsequent application of k-means clustering to categorize image tiles based on these features illustrates the synergy between CNNs and traditional data analysis methods, allowing the identification of meaningful correlations between urban characteristics and health outcomes.

Levy et al [41] explored the use of CNNs for predicting mortality rates from satellite images, demonstrating the models’ capability to discern spatial patterns related to health outcomes. By comparing DL models with linear regression and a hybrid approach, the study delineated CNNs’ efficiency in modeling complex, nonlinear relationships between environmental features and health indicators.

Guo et al [42] used Bayesian analysis and partial least squares regression in conjunction with the ResNet-50 architecture to examine obesity trends and the impact of environmental factors on obesity prevalence among Chinese children and adolescents. This integration of statistical methods with DL techniques demonstrates a comprehensive approach to understanding the multifaceted influences on obesity, offering predictive insights into spatial and temporal variations of health outcomes.

#### Semantic Segmentation for Urban Environment Analysis

Han et al [43] deployed SegNet for semantic segmentation of Google Street View images to study urban environments’ impact on psychological stress. SegNet, designed for pixel-wise segmentation, features an encoder-decoder architecture for contextual detail abstraction and precise urban element classification. This approach enabled detailed urban feature analysis, including buildings and green spaces, for assessing their influence on psychological stress. Using a combination of segmented imagery analysis within a human-machine adversarial framework and random forest classification, the study provided insights into visual urban characteristics’ effects on stress, demonstrating DL’s utility in urban health research.

Hong et al [44] used the U-Net architecture for semantic segmentation of unmanned aerial vehicle imagery to map green spaces and sidewalks, integrating DL with a GIS for urban and public health analysis. U-Net’s encoder-decoder design, enhanced with skip connections, excels in extracting detailed features from high-resolution unmanned aerial vehicle images, crucial for accurate environmental mapping at the neighborhood level. This method optimizes the segmentation process, distinguishing between various urban elements and facilitating the comprehensive analysis of green spaces and sidewalks. By leveraging U-Net for precise pixel classification, the study demonstrates DL’s efficacy in improving urban environment datasets, thus supporting targeted urban planning and public health initiatives.

In their analysis, Wang et al [45] applied the Fully Convolutional Network (FCN)–8s architecture to semantic segmentation of Tencent Street View (Tencent) images for studying the built environment’s effects on health in older adults within Beijing’s Haidian District. FCN-8s, a CNN that is fully convolutional and optimized for pixel-level segmentation, enabled the classification of urban features directly from images, facilitating the examination of variables, such as wealth, safety, and green space. The network uses convolution and deconvolution layers for processing spatial information, enhancing segmentation accuracy for complex urban scenes. This approach underscores the applicability of FCN-8s in urban analysis, providing a detailed assessment of environmental attributes and their correlations with health outcomes, thereby supporting data-driven urban planning and public health policy development.

Larkin et al [46] used GISs, satellite imagery, and a pyramid scene parsing network (PSPNet), designed for semantic segmentation, to analyze urban perceptions across 56 cities. PSPNet classifies each pixel in Google Street View images into urban elements (eg, trees, buildings, and roads) by leveraging a pyramid pooling module that aggregates context information at different scales, ensuring detailed scene parsing and global context comprehension. This method, combined with GIS-derived data on population density and green spaces, allowed for the quantitative linking of urban features to perceptions of safety, liveliness, and beauty. The study showcases the integration of DL with GISs, demonstrating PSPNet’s effectiveness in urban landscape analysis and the potential of merging image-derived metrics with environmental data for insights into the built environment’s impact on human perceptions.

#### Discriminative and Generative Models in Urban Health Studies

Newton detailed both discriminative and generative models within the framework of CNNs for obesity studies in urban contexts, as detailed in the chapter “Deep Learning in Urban Analysis for Health” of the book *Artificial Intelligence in Urban Planning and Design* [47]**.** This examination reveals DL’s capacity to identify subtle spatial patterns associated with health outcomes, reinforcing its value in enhancing urban planning and public health policy through advanced model applications.

#### Review Studies on ML and DL Applications

Siddiqui et al [48] offered a comprehensive review of ML and DL models for predicting childhood and adolescent obesity. This survey integrated 39 studies, examining a variety of ML models (including artificial neural network, recurrent neural network, and CNN) and datasets (ranging from surveys and cohorts to electronic health records and image datasets). The literature was categorized based on methodologies (traditional ML vs DL), dataset types, and outcomes predicted (overweight, obesity, or both). The survey distinguished between studies aimed at identifying risk factors and those predicting obesity, underlining the significance of model interpretability. It identified research gaps and advocated for advancements through large-scale multimodal datasets and the development of interpretable models for obesity prediction.

Wirtz Baker et al [49] conducted a systematic review under PRISMA (Preferred Reporting Items for Systematic Reviews and Meta-Analyses) guidelines to evaluate the use of nontraditional data sources, such as geospatial data and social media, in studying the built environment’s impact on obesity. Analyzing 53 studies, this review emphasized the value of geospatial data within GISs for assessing physical environment features and the insights social media data offer into sociocultural dynamics related to obesity. The review suggested future research avenues should leverage digital advancements to gain a comprehensive understanding of obesity’s environmental determinants.

Zhou et al [50] provided a mini-review focusing on the application of open-source ML models in obesity research. This review selected 25 studies, categorizing ML models into supervised and unsupervised learning and covering applications in nutrition, the environment, genetics, and the microbiome. The review aimed at research reproducibility and efficiency, advocating for open-source tools and the integration of diverse data sources for a multifaceted analysis of obesity’s causes.

Shaamala et al [51] presented a systematic review of ML applications in geospatial analysis, identifying key application areas including classification, detection, extraction, clustering, regression, modeling, prediction, and optimization. Techniques, such as random forests, support vector machines, and CNNs, were highlighted for their effectiveness in classification, detection, and extraction tasks, with specific mention of architectures such as U-Net and You Only Look Once for semantic segmentation and object detection. The review also discussed the use of ensemble models and advanced neural networks in regression, modeling, and prediction tasks, along with optimization algorithms for spatial planning. This work aimed to map the current landscape and identify future research directions in ML applications for geospatial analysis.

### Cross-Disciplinary Applications of Advanced ML

Recent advances in ML [52-54], highlight the potential of such technologies in both pandemic responses and chronic disease management like obesity. These studies used sophisticated algorithms— dilated efficient residual global attention, fuzzy ensemble models, and artificial intelligence techniques for minimal data analysis, to enhance diagnostic and predictive accuracies, akin to our use of DCNNs for analyzing environmental determinants of obesity from satellite imagery. The success of these methods in medical diagnostics underscores their applicability in spatial health studies, suggesting promising avenues for using similar approaches in obesity research to improve public health outcomes.

### Spatial Analysis and Geolocation

Spatial analysis and geolocation are pivotal tools in obesity research, providing crucial insights into the complex interplay between environmental factors and obesity prevalence. By leveraging these techniques, researchers can map and analyze spatial patterns of obesity, identify obesogenic environments, and understand how individual interactions with these environments contribute to obesity risk [55,56]. Through the integration of GISs, GPS, and RS, spatial analysis and geolocation enable the examination of geographical variations in obesity rates, the assessment of accessibility to health-promoting resources, and the evaluation of the impact of urban planning on physical activity levels. Consequently, they play an indispensable role in advancing our understanding of the spatial determinants of obesity and informing targeted interventions and policies to combat this global health challenge [55,57].

## Methods

### Overview

Our study analysis consisted of 4 main steps. First, we processed Sentinel-2 satellite images to extract features of the built environment using ResNet-50. Second, we merged the polygon’s census tracts using the TIGER (topologically integrated geographic encoding and referencing)/Line shapefiles with the obesity rates for each census tract using the US Centers for Disease Control and Prevention (CDC) data [58]. Third, we conducted an exploratory spatial analysis (ESA), particularly spatial autocorrelation and local clustering, to determine the modeling approach and techniques. Fourth, as our ESA suggests that there is substantial spatial autocorrelation and spatial dependency in the obesity rates, we used a spatial regression technique, particularly the spatial lag model (SLM), to build a model to predict the obesity rate for each census tract and to assess the association between the built environment and obesity prevalence.

**Figure 1:**
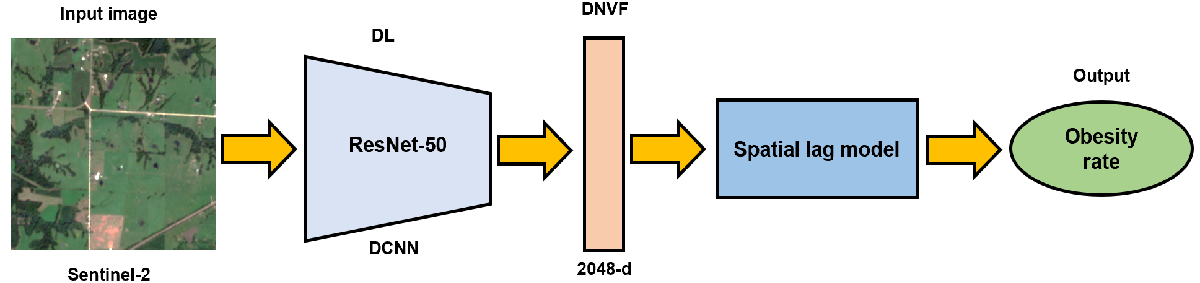
Flowchart illustrating the process of using Sentinel-2 satellite imagery processed through a Residual Network-50 (ResNet-50) deep convolutional neural network (DCNN) to extract deep neural visual features, which are then used as the inputs of the spatial lag model, ultimately yielding predicted obesity rates for geographic regions. DL: deep learning; DNVF: deep neural visual feature.

Figure 1 illustrates an analytical workflow designed to predict obesity rates from satellite imagery. The process begins with a high-resolution input image from the Sentinel-2 satellite, capturing the intricate details of the Earth’s surface. The image on the left in Figure 1 was raw data that was fed into a DL framework. Inside this framework, a ResNet-50 DCNN was used to analyze and extract high-dimensional features from the image, known as DNVFs, which are 2048D vectors encapsulating the critical visual cues from the input data. Subsequently, the extracted features were used as the input of the SLM, which finally output the predicted obesity rate of the given geographic unit. This output is represented as a quantifiable measure, providing valuable insights for public health and resource allocation.

### Obesity Prevalence Data

**Figure 2:**
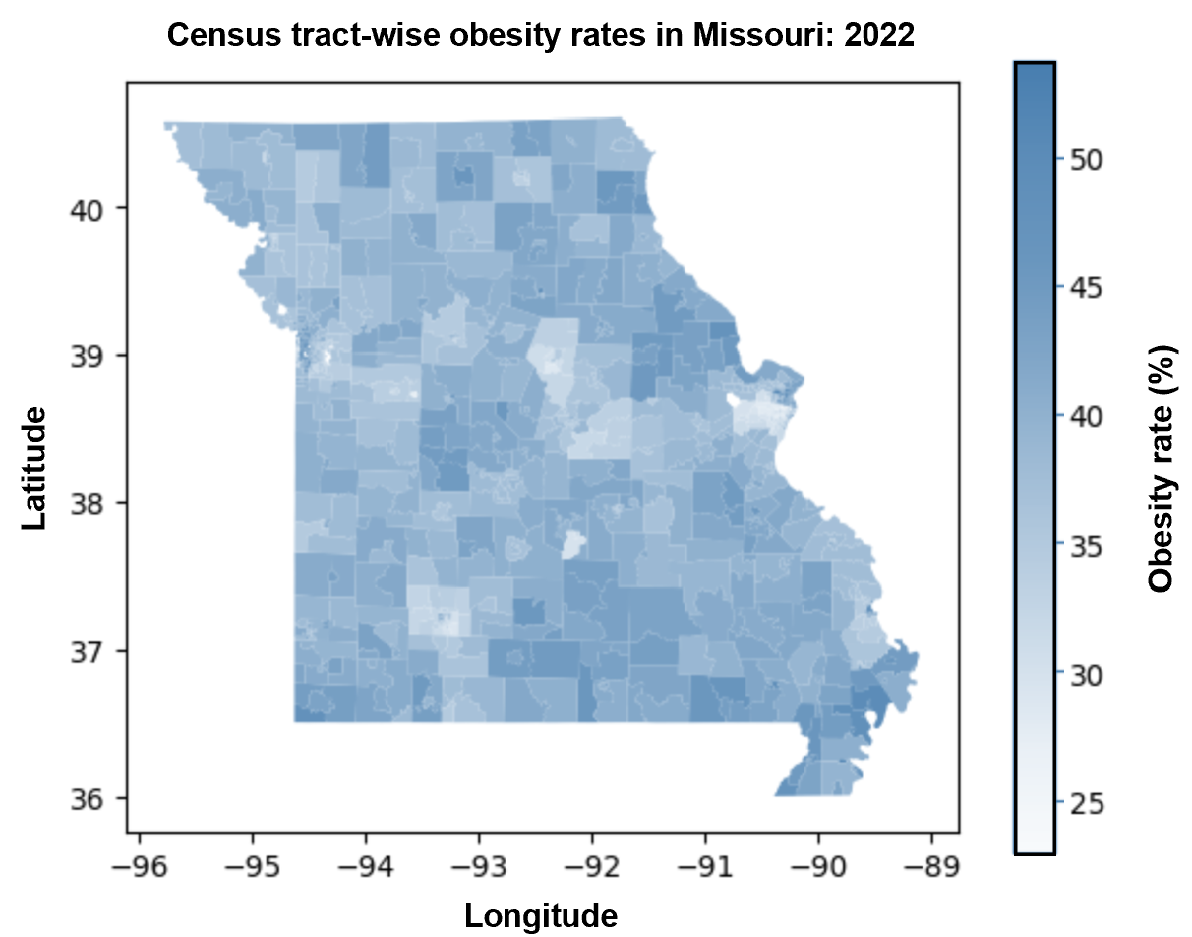
Choropleth map displaying the distribution of obesity rates for individuals across Missouri census tracts in 2022. The variations in the color intensity reflect the range of obesity prevalence, with darker blue indicating higher obesity rates. The color scale to the right quantifies the obesity rates corresponding to each color shade.

Figure 2 provides a choropleth map that serves as a visual representation of obesity prevalence among the various census tracts within the state of Missouri for the year 2022. Each census tract is color-coded to denote the obesity rate percentage for individuals residing in that tract, with the intent of offering a comprehensive geographic overview of the obesity landscape across the state. In the map, lighter shades of blue show lower obesity rates, and darker hues correspond to higher obesity rates. The scale itself delineates a range starting at 25%, represented by a light blue, and progresses to 50%, indicated by a dark blue color. Notably, the map reveals a significant variation in obesity rates across different regions, with some census tracts exhibiting markedly higher rates and others reflecting lower obesity rates.

We used 2022 estimates of annual crude obesity prevalence at the census tract level, derived from the 500 Cities project [59,60]. These estimates are based on data from the Behavioral Risk Factor Surveillance System, which surveys individuals aged ≥18 years [60]. Obesity is identified using a BMI threshold of 30, calculated as the individual’s weight in kg divided by their height in m^2^ [59]. Our study focused on the mid-Missouri region in the United States. The 1052 census tracts (Missouri State) covered in this study have an aggregate area of 69,707 square miles (180,540 km^2^). They have a total population of 6.2 million (based on the 2020 census).

The CDC data list 1387 census tracts, 4506 block groups, and 343,565 census blocks. Given that the number of TIGER/Line census tract shapefiles in the state was 1654, polygon (census tract) IDs in the TIGER/Line data had to be aligned to census tract IDs in the CDC data. To fix the mismatching issue, census tracts with subdivisions (tract names with 2 trailing digits different from 0) in both datasets were joined into larger polygons.

First, we joined the polygons in the TIGER/Line dataset by removing all subdivisions.

Of the initial 1654 polygons, 55.26% (n=914) had names following a naming convention of the type “XXXX.YY,” where “YY” corresponded to the subdivision within a particular tract. The remaining 44.74% (n=740) had no subdivisions and names containing 2 trailing zeros (eg, “XXXX.00”). Setting the 2 trailing digits to 0 in these 914 names resulted in repeated names with 323 unique names. If a name was repeated, all repeated elements were joined to become a single polygon, which resulted in a set of 1063 polygons.

Similarly, the IDs of the census tracts in the CDC data consisted of a string of digits, with the last 2 digits corresponding to a set of subdivisions different from that of the TIGER/Line data. Of the 1387 tracts, 63.52% (n=881) had IDs with 2 trailing zeros (no subdivisions), while the remaining 36.48% (n=506) had subdivisions. We set the initial 506 string IDs to a single subdivision with repeated entries, which resulted in 178 unique IDs. Because each of these new unique IDs had multiple obesity rates, an average obesity rate was calculated as a proxy for the obesity rate of the newly joined area. The average obesity rate for these joint areas, weighted by census tract population, was calculated as follows:



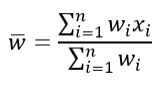



where 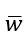 is the new obesity rate for the joint area, *n* is the number of subdivisions (repeated entries) within the original census tract, *w_i_* is the population in a subdivision *i*, and *x_i_* is the obesity rate in *i*. The joined 1059 CDC entries were then matched to the joined 1063 TIGER/Line tracts, for a final overlapping set of 1055 census tract polygons with their corresponding obesity rates, which we used as the inputs to our models.

### Acquiring Satellite Imagery

We acquired our imagery inputs by selecting the Sentinel-2 products intersecting our previously defined set of census tracts. These satellite products were downloaded from ESA’s Copernicus Dataspace Ecosystem. Because ESA’s OpenSearch application programming interface uses http requests to search for products, we defined a shortened geometry string that could fit in our search query. This was done by joining our previously defined census tract polygons into a state boundary that was further simplified into a closed polygon of 54 vertices using the implementation of the Douglas-Peucker algorithm included in GeoPandas.

**Figure 3:**
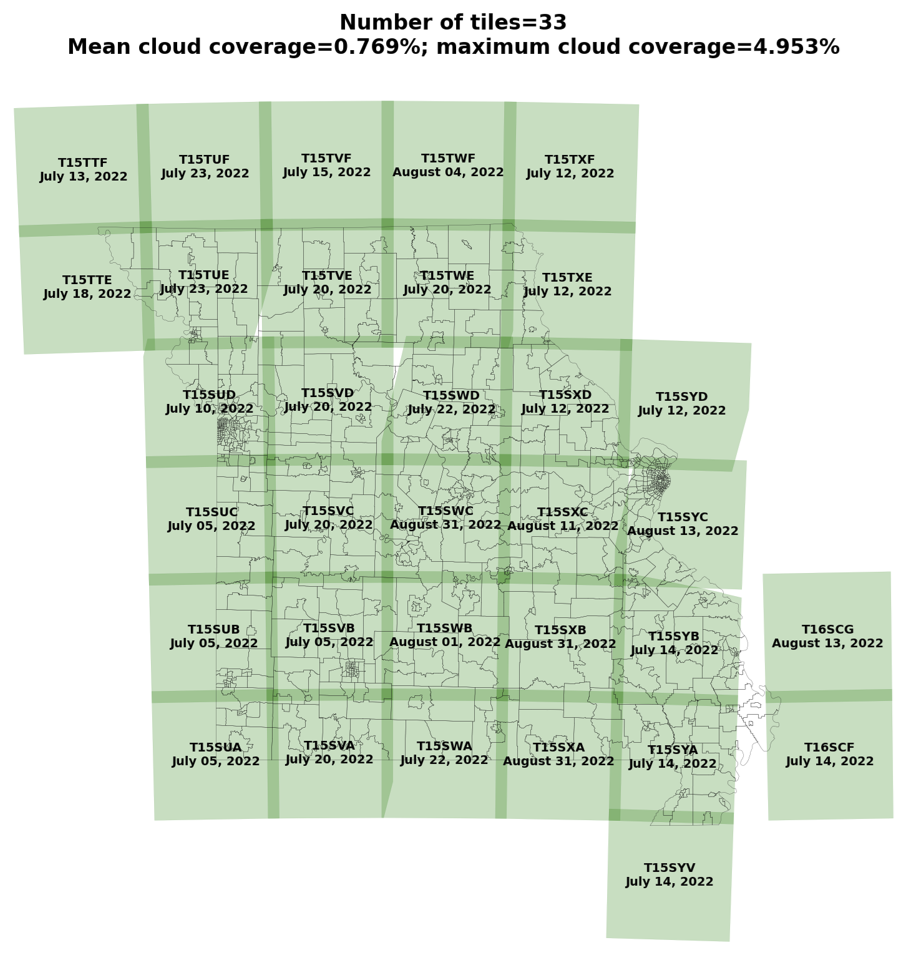
A chart illustrating the collection of 33 Sentinel-2 satellite image tiles, each identified by a specific tile code and date of capture in 2022. The chart provides information on cloud cover across these images, with a mean cloud coverage of 0.769% and a maximum cloud coverage of 4.953%.

Figure 3 presents our geospatial imagery coverage data analysis using 33 Sentinel-2 satellite images of Missouri from the year 2022, each with a resolution of 10,980×10,980 pixels at 10 m per pixel.

Our search resulted in 187 intersecting Sentinel-2 products between July 1, 2022, and August 31, 2022. Overlapping images were removed in 2 steps. First, products with completely overlapping geometries (corresponding to the same universal transverse mercator zone tile) were filtered by discarding all but the product with the largest area and the lowest cloud percentage. Second, 7 partially overlapping products (which also happened to have little state coverage) were discarded after visual inspection. This resulted in a set of 33 Sentinel-2 images that were used to define our model inputs. These products were downloaded from ESA’s Dataspace Ecosystem to Nautilus. All 33 Sentinel-2 image sizes were 10,980×10,980 pixels. The images were then normalized and tiled into 224×224 pixel chips. This created 82,500 three-band (red-green-blue) image chips.

### Image Processing

#### Overview

Building on acquiring Sentinel-2 imagery, our image-processing efforts used DCNNs to advance the analysis of these extensive datasets [61,62]. We used the DNVFs from a pretrained neural network to obtain the built area features of the 82,500 (63,592 intersected with Missouri census tracts) satellite image chips. We used the ResNet-50 network, which is composed of 50 layers (48 convolution layers along with 1 max pool and 1 average pool layer) and is trained on approximately 1.2 million images from the ImageNet database (a dataset of >14 million images used for large-scale visual recognition challenges) for recognizing objects belonging to 1000 categories [63]. For each chip we passed through the network, we extracted the 2048 features from the last hidden layer of the network before the output layer [64]. Because each census tract in our dataset could have >1 image chip intersecting it, we calculated a corresponding weighted mean feature vector for a tract with the features of the intersecting chips [65]. For a census tract *t*, its mean feature vector *F_t_* was calculated as follows:



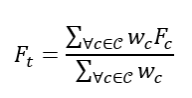



where *C* is the set of image chips intersecting the census tract, *F_c_* is a ResNet-50 feature vector obtained from chip *c,* and *w_c_* is the (scalar) number of pixels in *c* intersecting the census tract. To include the obesity rate of a given chip in our analysis, the inverse case was considered. When a chip intersected with >1 census tract, we calculated the obesity rate in such a chip as the average obesity rate of all the intersecting census tracts [65]. So, for a chip *c,* the weighted obesity rate is similarly calculated as follows:



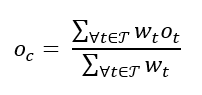



where *T* is the nonempty set of tracts intersecting a chip *c*, *o_t_* is the obesity rate in a census tract, and *w_t_* is the number of pixels in *c* corresponding to the *t* census tract. We do not link these features to specific elements in the built environment or the obesity prevalence. Rather, these DNVFs collectively represent an indicator to help predict the obesity prevalence for each census tract. The image chips were tiled for a size of 224×224 pixels or 2240 m (2.24 km) by 2240 m (2.24 km). The graphics processing unit (NVIDIA GeForce RTX 2080 Ti)–accelerated processing speed was approximately 77.51 tiles per second or 1064.25 seconds (17.73 min) of DCNN inference for our study area. This translates to a rate of 0.0129 seconds per tile.

#### Spatial Autocorrelation

Following image processing, we explored spatial autocorrelation to understand the interdependencies of obesity rates across different geographic locales. Spatial autocorrelation is a concept in spatial analysis that quantifies the degree of similarity between observations in neighboring geographic locations. It measures how much the value of a variable at one location is influenced by the values of the same variable at nearby locations. Positive spatial autocorrelation indicates that similar values are clustered together, while negative spatial autocorrelation suggests that dissimilar values are adjacent to each other. This concept is crucial in identifying patterns and relationships in geographic data, as it helps to determine whether the spatial distribution of a variable is random, clustered, or dispersed [66,67]**.**

In obesity research, spatial autocorrelation is used to examine the spatial distribution of obesity rates or related factors, such as physical activity levels, access to healthy foods, or socioeconomic status. By assessing the degree of spatial autocorrelation, researchers can identify areas where obesity rates are higher or lower than expected based on the surrounding locations. This analysis can reveal clusters of high or low obesity prevalence, indicating potential hot spots or areas of concern. It also helps to understand the spatial dynamics of obesity, which can inform targeted public health interventions and policies [67,68]**.**

The application of spatial autocorrelation in obesity research extends to exploring the environmental and social determinants of obesity. By analyzing the spatial autocorrelation of variables, such as access to parks, fast-food outlets, or income levels, researchers can investigate how these factors contribute to the spatial variation in obesity rates. This can lead to a better understanding of the complex interactions between the built environment, socioeconomic factors, and obesity, ultimately guiding the development of more effective strategies for obesity prevention and management at the local and regional levels [67-69].

#### Global Moran I

To quantitatively measure the spatial autocorrelation observed, we applied global Moran I, a statistical tool that evaluates the correlation of a variable with its spatially lagged counterpart. It is a widely used tool in spatial analysis, especially in fields such as geography, ecology, and epidemiology [70,71]**.**

Moran I is defined as the correlation coefficient between a variable and its spatially lagged counterpart. It measures the extent to which similar values of a variable are located near each other in space [70,72]**.**

The global Moran I is calculated as follows:



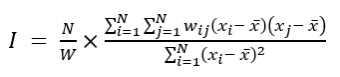



where *N* is the number of spatial units and *w_ij_* is the spatial weight between units *i* and *j*. *x_i_* and *x_j_* are the values of the variable of interest at units *i* and *j*, respectively. 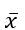 is the mean value of the variable across all spatial units. *W* is the sum of all spatial weights, 
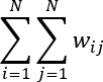
.

Moran I is a crucial measure in spatial analysis, used to identify and quantify spatial patterns in a dataset. A positive Moran I indicates spatial clustering, where areas with similar values are geographically close to each other. For instance, regions with high values tend to be surrounded by other high-value regions, and the same applies to low values. In contrast, a negative Moran I suggests spatial dispersion, where areas with high values are typically surrounded by areas with low values, indicating a dissimilar distribution. When Moran I is close to 0, it implies a random spatial pattern, signifying no significant spatial autocorrelation among the observed values [70-72]**.**

In our research, we focused exclusively on Moran I as our measure of spatial autocorrelation, opting not to use Geary C. While Geary C is sensitive to local variations and emphasizes the dissimilarity between neighboring observations, Moran I provides a more comprehensive view of the overall spatial pattern. To assess the statistical significance of Moran I, we used permutation tests or analytical methods to create a reference distribution under the null hypothesis of no spatial autocorrelation. By comparing the observed Moran I to this distribution, we can determine its significance and thereby gain valuable insights into the spatial relationships and processes at play in our study [70-72]**.**

#### Local Indicators of Spatial Association

Further dissecting spatial patterns, we used local indicators of spatial association (LISA) statistics to pinpoint specific areas exhibiting significant spatial clustering or dispersion. Unlike global measures, such as Moran I, which provide a single summary statistic for the entire study area, LISA statistics offer insights into the spatial patterns at individual locations, allowing for the detection of local clusters or outliers [73,74]**.**

LISA statistics are designed to assess the degree of spatial autocorrelation for each observation within a dataset, considering the values of neighboring observations. They help to identify areas where the value of a variable is significantly different from or similar to its surrounding values, indicating the presence of spatial clusters or hot spots [75,76]**.**

LISA statistics play a pivotal role in spatial analysis by providing a detailed examination of local spatial patterns within a dataset. These statistics are instrumental in detecting local clusters of high or low values, commonly referred to as hot spots or cold spots, which can reveal areas of concentrated phenomena, such as high crime rates or regions of environmental degradation [76,77].

In addition, LISA statistics are adept at identifying spatial outliers, where the value of an observation significantly deviates from the values of its neighboring observations, indicating anomalies or irregularities in the spatial distribution. By exploring the local spatial patterns and relationships, LISA statistics offer a nuanced understanding of the spatial dynamics at play, allowing researchers to uncover subtle variations and trends that may be obscured in a global analysis. This localized approach is essential for targeted interventions and policy making, as it provides a granular view of the spatial structure of the data, enabling more precise and effective responses to spatially varying challenges [74,75,77]**.**

One of the most commonly used LISA statistics is local Moran I. The local Moran I for observation *i* is calculated using the following equation:



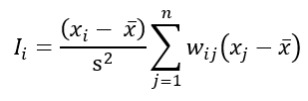



where *I_i_* is the local Moran *I* for observation *i*. *x_i_* and *x_j_* are the values of the variable for observations *i* and *j*, respectively. 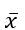 is the mean value of the variable across all observations. s^2^ is the variance of the variable. *w_ij_* is the spatial weight between observations *i* and *j*. *n* is the total number of observations.

The local Moran I statistic is used to assess the degree of spatial autocorrelation at the local level, identifying clusters of similar values and spatial outliers.

In assessing the significance of LISA statistics, permutation tests are commonly used. This involves randomly shuffling the observed values and recalculating the statistic multiple times to generate a reference distribution. By comparing the observed LISA values to this distribution, researchers can determine the statistical significance of the spatial patterns detected. This process is crucial for ensuring that the identified clusters and outliers are not due to random chance but are indicative of underlying spatial processes [74,77]**.**

In the context of obesity research, LISA statistics provide invaluable insights into the local spatial dynamics of obesity prevalence and related factors. High positive values of Local Moran I can reveal clusters of areas with high obesity rates (high-high clusters), while high negative values can indicate regions where high obesity rates are surrounded by areas with lower rates (high-low outliers), and vice versa [73,76].

By visualizing these patterns on maps, researchers can identify specific neighborhoods or regions that may require targeted public health interventions. Moreover, the detailed local insights offered by LISA statistics can aid in understanding the spatial distribution of obesity and its association with environmental and socioeconomic factors, ultimately informing more effective strategies for addressing the obesity epidemic at a granular level [77]**.**

#### Incorporating Spatial Dependencies With an SLM

Integrating the insights gained from both global and local spatial analyses, we used the SLM to incorporate the spatial dependencies identified among the observations into our regression analysis. It is used to account for the influence of neighboring observations on each other, which is a common phenomenon in spatial data [78]**.**

In an SLM, the dependent variable in one location is assumed to be affected by the values of the dependent variable in neighboring locations. This spatial dependence is captured by including a spatially lagged dependent variable as an additional explanatory variable in the regression model [79]**.**

SLMs are used in various fields, including economics, geography, and public health, to analyze spatial data where observations are not independent but influenced by nearby observations. It helps to provide more accurate estimates and inferences by accounting for spatial dependency [80]**.**

In obesity research, SLMs can be used to study the spatial distribution of obesity rates and their determinants. For example, it can be used to examine how obesity rates in one area are influenced by the rates in adjacent areas, which might be due to shared environmental factors, social networks, or economic conditions. This can provide insights into the spatial diffusion of obesity and help in identifying areas that might benefit from targeted interventions [78-80]**.**

The SLM is represented by the following equation:



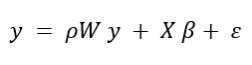



where *y* is the vector of the dependent variable (eg, obesity rates). *ρ* is the spatial autoregressive parameter, representing the strength of the spatial dependence. *W* is the spatial weight matrix, defining the spatial relationship between observations. *X* is the matrix of independent variables (eg, socioeconomic factors and access to healthy food). *β* is the vector of coefficients for the independent variables. *ε* is the vector of error terms.

In this model, ρ*W* y is the spatially lagged dependent variable, capturing the influence of neighboring observations on each observation in the dataset. In the SLM, the spatial weight matrix *W* is essential for capturing the spatial correlations between nearby observations. We computed *W* using the contiguity method, more specifically the “queen” case, which is best suited for polygon features as it defines neighbors based on shared boundaries or vertices between spatial units (eg, census tracts). This approach identifies neighboring spatial units by determining which share at least 1 point of contact, either a border or a corner. Once the queen contiguity structure was defined, the weights were standardized so the sum of weights for each observation equals 1, ensuring comparable influence across all spatial units of analysis.

The estimation of the SLM typically requires specialized techniques, such as maximum likelihood or spatial 2-stage least squares, due to the presence of the spatially lagged dependent variable. The SLM is a powerful tool in spatial analysis, particularly valuable in fields, such as obesity research, where the spatial distribution of variables is of interest. By accounting for the influence of neighboring observations, the SLM provides a more accurate representation of spatial dependencies, leading to better-informed decisions and interventions.

### Ethical Considerations

We did not seek further approval or exemption from an IRB because this study falls under Exemption 4 of the NIH Human Subjects Research Exemptions. This exemption applies as our study involved secondary analysis of an existing, IRB-approved CDC dataset [60], which is deidentified and publicly available, hence not requiring informed consent.

## Results

Table 1 presents a detailed summary of key metrics across various regions, such as population, area (in km^2^), obesity crude prevalence (%), and the number of chips. The data are systematically organized to illustrate the minimum, median, and maximum values for each metric, providing a clear understanding of the range and central tendencies within the dataset. For population, the minimum value recorded is 102, the median is 4058, and the maximum reaches 75,569, reflecting the diversity in population sizes across different areas. The area of the regions varies significantly, with the smallest being just 0.49 km^2^, the median at 20.19 km^2^, and the largest extending to 1787.47 km^2^. In terms of obesity rates, the lowest rate observed is 23%, the median stands at 39.2%, and the highest rate is 53.7%, indicating varied health metrics across the regions. Finally, the number of chips ranges from a minimum of 1 to a maximum of 442, with a median of 14, highlighting different levels of chip distribution or consumption.

**Table 1:** A summary of key regional metrics, covering total population, area (km^2^), obesity crude prevalence, and number of chips intersected with each tract.

	Values, median (range)
Total population, n	4058 (102-75,569)
Area (km^2^)	20.19 (0.49-1787.47)
Obesity crude prevalence (%)	39.2 (23-53.7)
Chips, n	14 (1-442)

**Figure 4:**
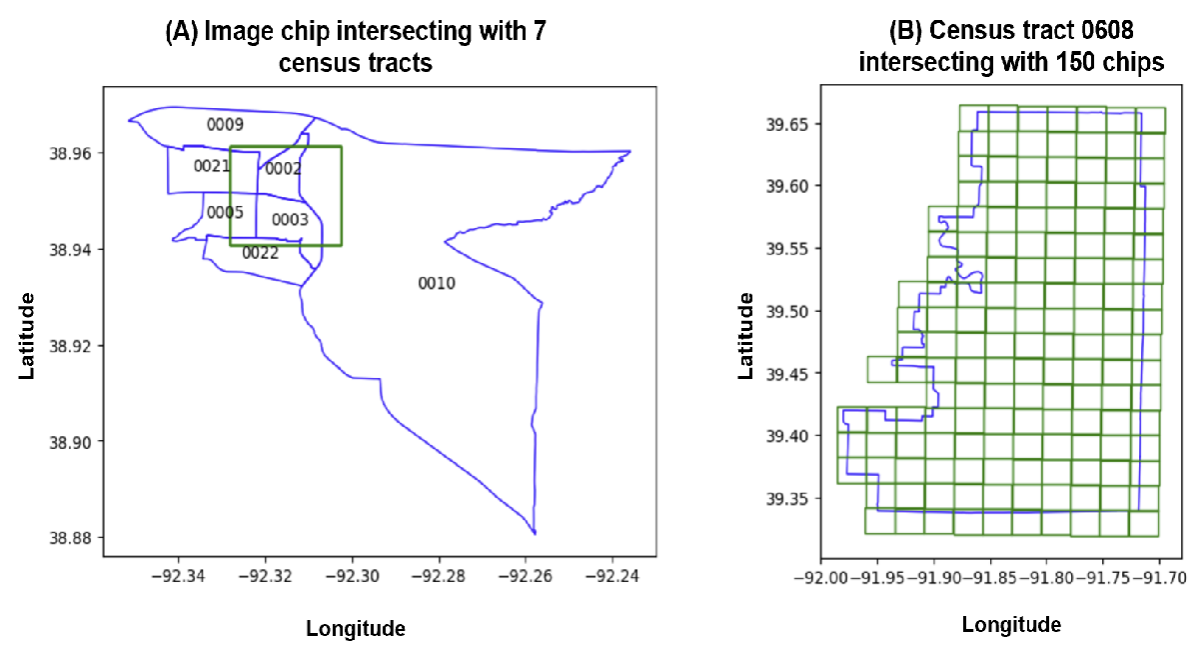
Multiscale analysis of satellite image chips and census tracts in Missouri.

Figure 4A details individual image chip boundaries, illustrating their overlap with 7 distinct census tracts (numbered for reference). Figure 4B further narrows down to census tract 0608, demonstrating the intersection with 150 specific image chips for granular analysis. The figure highlights the granularity and density of data distribution within the geographic study area.

Figure 4A displays a visualization of an individual image chip intersecting 7 distinct census tracts, enabling a detailed analysis of these specific overlaps. This image chip intersects with one of the highest numbers of census tracts (0002, 0003, 0005, 0009, 0010, 0021, and 0022), and it is located in Boone County. Figure 4B is a single census tract, specifically “Census Tract 0608” with 150 intersecting image chips. This census tract is among the areas with one of the highest numbers of image chips. This level of granularity reveals a concentrated cluster of data points, potentially signifying a region of particular interest or higher measurement intensity.

**Figure 5:**
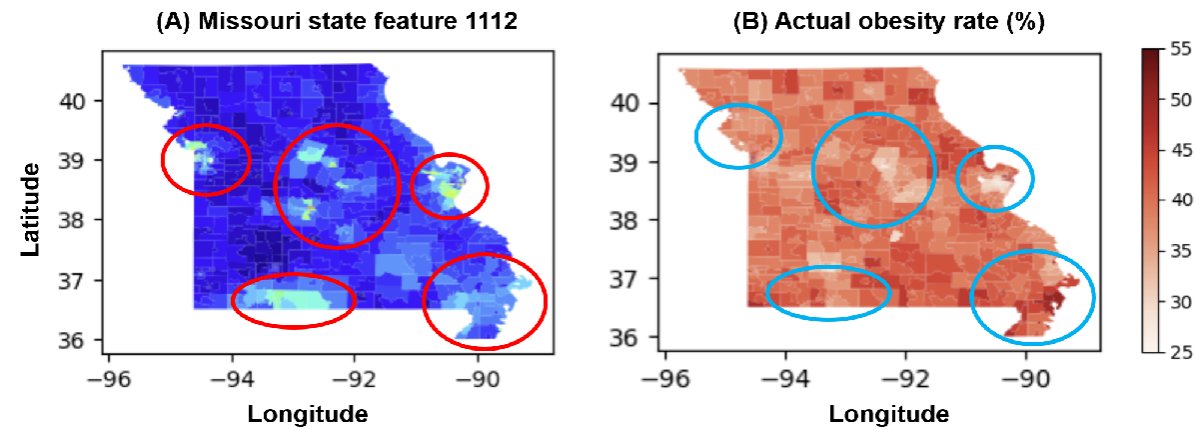
A map showing feature 1112 (left) values across census tracts in Missouri compared with the obesity rate (right).

The left map in Figure 5 depicts the spatial distribution of feature 1112 across Missouri, with red circles highlighting areas of high values, which seems to correlate with (urban areas). The right map depicts actual obesity rates (%) across the state, with blue circles indicating regions with lower obesity prevalence. Notable discrepancies between feature values and obesity rates can be observed in several regions.

**Figure 6:**
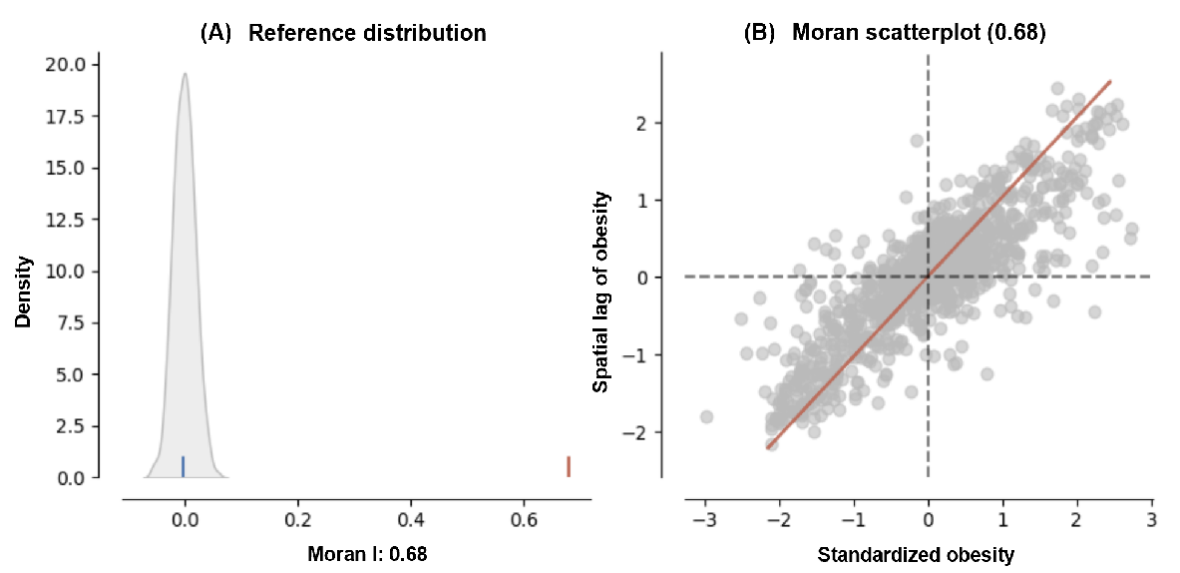
(A) Reference distribution for Moran I, confirming positive spatial autocorrelation with an observed value of 0.68, significantly deviating from the null hypothesis of spatial randomness (blue line). (B) Moran scatterplot, demonstrating the relationship between standardized obesity rates and their standardized spatial lag, with a clear positive slope indicating that regions with high obesity rates are typically surrounded by similar regions. These findings are instrumental for spatially informed public health strategies, pinpointing areas for targeted interventions and enhancing the precision of predictive models for obesity prevalence across the state.

Figure 6A is a visualization of a probability distribution related to the Moran I statistic, a measure used in spatial analysis. The bell-shaped curve represents the expected distribution of Moran I under the null hypothesis of spatial randomness. The x-axis shows the range of Moran I values, and the y-axis represents the density or the probability of those values. There is a vertical line marked on the plot that indicates the observed Moran I value, which here is 0.68. This is significantly higher than 0, suggesting that there is a significant positive spatial autocorrelation in the dataset. The peak of the distribution curve is centered very close to 0, with the tails tapering off smoothly on either side. A blue line marks the mean of the reference distribution, indicating where Moran I would fall if the null hypothesis were true (no spatial autocorrelation). A red line marks the actual observed Moran I, which falls far to the right of the mean, indicating a stronger-than-expected positive spatial correlation.

Figure 6B depicts a scatterplot with a regression line. This scatterplot is used to visualize the spatial autocorrelation of a variable, here, obesity rates. The x-axis represents standardized values of obesity rates, meaning the data have been normalized to have a mean of 0 and an SD of 1. The y-axis represents the standardized spatial lag of obesity, which is essentially the average obesity rate of neighboring areas. The scatterplot is dense with points that represent the different locations (such as census tracts) within Missouri. The points are predominantly clustered around the regression line (red line), suggesting that areas tend to be similar to their neighbors’ high values are next to high values, and low values are next to low values.

In addition, the regression line slopes upward, reinforcing the indication of positive spatial autocorrelation, as higher standardized values of obesity are associated with higher spatial lags.

**Figure 7:**
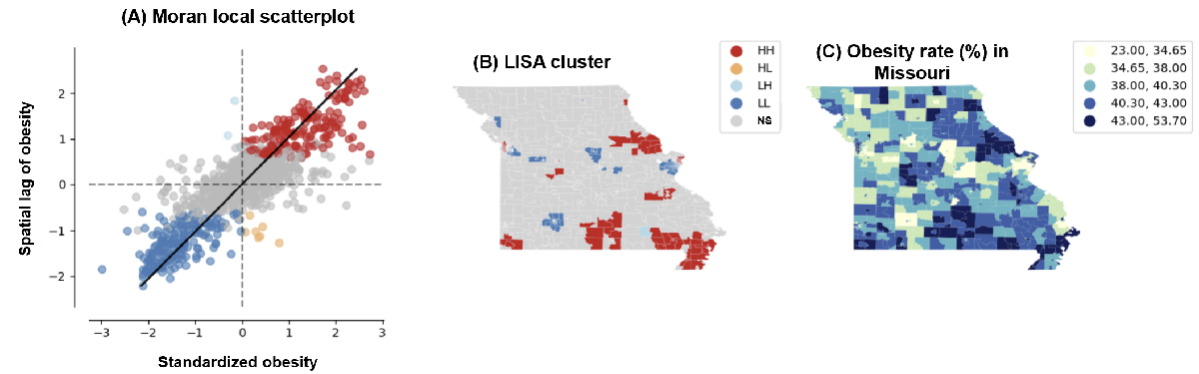
Multifaceted spatial analysis of obesity rates in Missouri using deep learning and satellite imagery. (A) Moran local scatterplot, indicating spatial autocorrelation of obesity rates with clusters of high (high-high; HH) and low (low-low; LL) values, alongside substantial spatial outliers (low-high; LH and high-low; HL). (B) Map of the local indicators of spatial associations (LISA) clusters across Missouri, identifying regions with statistically substantial spatial associations of obesity rates. (C) Actual obesity prevalence (illustrated with a choropleth map), correlating geographic data with obesity percentages. Combined, these visual tools leverage deep neural visual features extracted via a Residual Network-50 (ResNet-50) model applied to Sentinel-2 satellite imagery, offering a robust framework for understanding and predicting obesity distribution at the community level. NS: not significant.

Figure 7A represents a local version of Moran I scatterplot, which examines the spatial autocorrelation of obesity rates at a localized level. The x-axis displays the standardized obesity rates, indicating the deviation from the mean rate of obesity. The y-axis shows the spatial lag of obesity, which represents the average rate of obesity in neighboring locations. Points are color coded; blue represents low-low clusters where locations and their neighbors have lower than average obesity rates; red represents high-high clusters where locations and their neighbors have higher than average rates; tan points represent low-high or high-low outliers where a location’s obesity rate significantly differs from its neighbors. The solid black line indicates the trend, and the presence of color-coded points away from the origin suggests spatial clusters and outliers.

Figure 7B provides a spatial representation of LISA results, showing clusters and spatial outliers of obesity rates across Missouri. The map of Missouri is marked with areas color coded consistent with the legend in Figure 7A, with high-high clusters in red and low-low clusters in blue, among others. This visualization allows for the geographical identification of areas with statistically significant local spatial autocorrelation, revealing regions where similar or dissimilar obesity rates cluster spatially.

Figure 7C depicts a choropleth map indicating the obesity rate by county or census tract across Missouri. The color gradient represents varying rates of obesity, with darker colors indicating higher rates. This visualizes the actual obesity prevalence geographically, which can be cross-referenced with the LISA results for a more nuanced understanding of spatial patterns.

As we delve deeper into the intricacies of spatial data, it is also essential to explore other spatial regression techniques, such as geographically weighted regression (GWR). GWR extends the capabilities of traditional spatial models by allowing relationships to vary across space, offering a nuanced understanding of local variations in the data. This progression from global to local models, from SLM to GWR, signifies a move toward more refined and location-specific analyses in spatial research.

**Figure 8:**
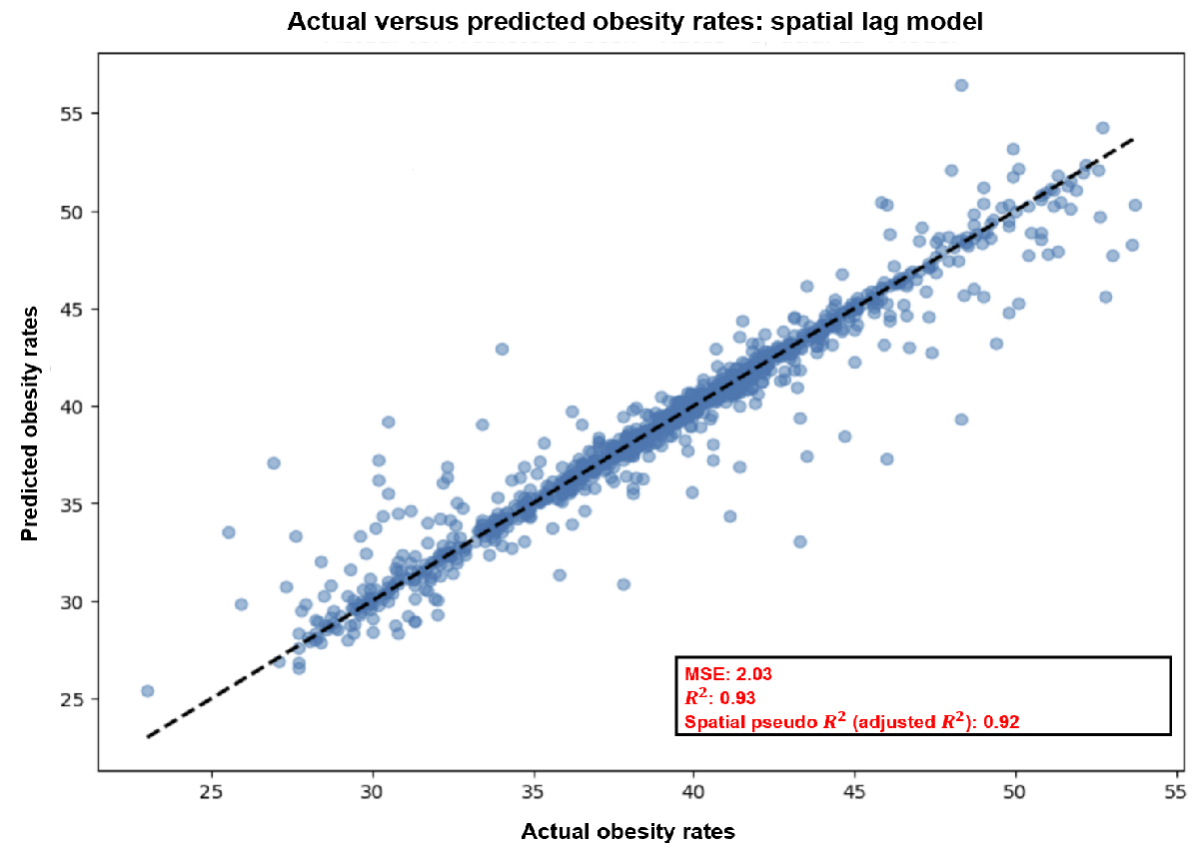
A scatterplot comparing actual versus predicted obesity rates in Missouri, derived from a spatial lag model using Residual Network-50 (ResNet-50) deep neural features and Sentinel-2 imagery. Data points close to the dashed best-fit line, with an R² of 0.93 and spatial pseudo R² of 0.92, highlight the model’s high accuracy in mapping the spatial distribution of obesity rates. MSE: mean squared error.

Figure 8 represents a scatterplot that compares the actual obesity rates with those predicted by a SLM, illustrating the model’s performance in estimating obesity rates across Missouri. The graph is a common tool used in regression analysis to validate the effectiveness of predictive models. The x-axis of the scatterplot displays the actual obesity rates, expressed as percentages, observed within the dataset. The y-axis, similarly, shows the obesity rates as predicted by the SLM. Each point on the graph represents a specific location within Missouri, such as a county or census tract, where the coordinates correspond to the actual and predicted obesity rates. The scatter of points is densest along the line of best fit, which is depicted by a dashed line, indicating a strong correlation between predicted and actual values.

The x-axis displays the actual obesity rates, expressed as percentages, observed within the dataset. The y-axis, similarly, shows the obesity rates as predicted by the SLM. Each point on the graph represents a specific location within Missouri, such as a county or census tract, where the coordinates correspond to the actual and predicted obesity rates. The scatter of points is densest along the line of best fit, which is depicted by a dashed line, indicating a strong correlation between predicted and actual values.

Beneath the scatterplot, 3 key performance indicators provide quantitative assessments of the model’s accuracy. The mean squared error, given as 2.03, is a measure of the average of the squares of the errors, the differences between predicted and actual rates. The lower the mean squared error, the better the model’s predictions match the observed data. The *R*^2^ value, noted as 0.93, represents the proportion of variance in the obesity rate that is predictable from the independent variables included in the model. An *R*^2^ of 0.93 suggests that 93% of the variability in the actual obesity rates can be explained by the model, which is a very high level of explanatory power. The spatial pseudo *R*^2^, which is the adjusted *R*^2^ for spatial regression models, is 0.92, reinforcing the model’s strong predictive capability while accounting for spatial dependencies.

Our study leveraged SLMs and DCNNs to predict obesity prevalence across 1052 census tracts in Missouri, achieving an *R*^2^ value of 0.93 and a spatial pseudo *R*^2^ of 0.92. These metrics indicate that our models explained 93% of the variability in obesity rates, demonstrating high predictive accuracy and robustness in modeling obesity prevalence using geospatial and DL methodologies.

In contrast, a similar study by Hales et al [25] used Visual Geometry Group-CNN-F models to analyze obesity across 1695 census tracts in 6 cities, achieving varied results across the different regions. Their overall *R*^2^ for the combined cities was lower, explaining 64.8% of the variation in obesity prevalence with a root mean square error of 4.3. Notably, the highest individual city *R*^2^ was 73.3% for Memphis, which is significantly lower than our study’s performance. Their study also highlighted a weaker performance when using point of interest data alone, which explained only 42.4% of the variation in obesity prevalence, with a root mean square error of 4.3 across all census tracts.

Our study’s superior performance is due to our use of a SLM to handle spatial dependencies, using ResNet-50 for advanced feature extraction from Sentinel-2 imagery, and focusing on Missouri for a more tailored geographic analysis.

## Discussion

### Principal Findings

The results in Figure 6 and the Moran I scatterplot can help spatially explain and predict obesity rates in Missouri using the DNVFs by identifying clusters of areas with either high or low rates of obesity. The positive Moran I value (0.68) suggests that similar values are located near each other. This means that if a certain census tract has a high obesity rate, it is likely that the neighboring tracts also have high obesity rates and vice versa for low rates. This pattern of spatial clustering is crucial for public health planning because it can help identify hot spots where interventions might be more necessary. For predictive modeling, spatial autocorrelation needs to be considered to improve the accuracy of predictions for obesity rates. Spatial models, such as SLMs or GWR, can use this autocorrelation to better understand and predict how obesity rates vary across Missouri. These models can incorporate not just the obesity rate of one area but also the context provided by surrounding areas, which can significantly influence health outcomes.

The subparts shown in Figure 6 help explain and predict obesity rates in Missouri. The Moran I scatterplot in Figure 7A identifies not only regions with high or low obesity rates but also those that deviate from the surrounding trend, which are crucial for targeted public health interventions. The LISA map in Figure 7B provides an immediate visual understanding of the geographic clustering of obesity rates, highlighting areas where policy interventions or further research might be needed. Finally, the choropleth map in Figure 7C allows researchers to observe the actual prevalence of obesity and how it correlates with the clusters identified through Moran I and LISA analyses.

These results, when combined with DNVFs extracted from the ResNet-50 model using Sentinel-2 satellite imaging, can enhance predictions and explanations of obesity rates. The ResNet-50 model, pretrained on ImageNet, can detect and analyze environmental and physical features that correlate with obesity rates. By integrating these features with spatial statistics, researchers can develop more sophisticated models that account for both the physical characteristics of the environment as captured in satellite imagery and the spatial relationships of obesity rates across the state. This multimodal approach allows for a deeper understanding of the drivers behind obesity patterns, which can inform more targeted and effective public health strategies.

The high *R*^2^ and adjusted *R*^2^ values in Figure 7 indicate that the SLM, which includes deep neural features extracted from the ResNet-50 model using Sentinel-2 satellite imaging, is highly effective in both explaining and predicting obesity rates. By incorporating the spatial lag of obesity rates, essentially considering not just the individual obesity rate of each area but also the influence of adjacent areas, the model captures the spatial autocorrelation inherent in the data. This consideration is crucial because the prevalence of obesity can be influenced by both location-specific factors and the characteristics of neighboring regions.

The use of DNVFs from ResNet-50 means that the model is leveraging complex, high-level features extracted from satellite imagery that may correlate with environmental factors affecting obesity rates, such as the availability of green spaces or the walkability of neighborhoods. The model’s success, as indicated by the scatterplot, demonstrates the potential for these advanced ML techniques to capture and use subtle spatial patterns and characteristics that contribute to public health outcomes.

Overall, the results depicted in this figure underscore the model’s potential as a tool for public health officials and policy makers. The ability to accurately predict obesity rates at a granular spatial level can facilitate targeted interventions and resource allocation, contributing to more effective public health strategies and better health outcomes for communities across Missouri.

### Limitations

Despite the promising results, our obesity research study has some limitations. First, the estimates of obesity prevalence from the Behavioral Risk Factor Surveillance System rely on self-reported measurements of height and weight, which are subject to bias and often result in an underestimation of the true rate of obesity [20,22]. Moreover, BMI fails to directly measure body fat, which can differ based on gender, age, race, and ethnicity. In addition, the risks of mortality and morbidity at a given BMI may not be the same across various racial and ethnic groups [23,24]**.** Variations in the timing between when the obesity data and the satellite images are collected can also introduce biases into our analysis.

One of the primary limitations of our study pertains to the dataset’s size and geographical coverage. The research was confined to 1052 census tracts within the entire state of Missouri, limiting the generalizability of the findings. Although these tracts were selected to represent a diverse range of urban and rural areas, they do not encompass other neighboring states or the entire country’s varied demographic and geographic profiles. Furthermore, the limited number of census tracts might not provide a sufficiently robust dataset for more complex ML models. The use of Sentinel-2 imagery, while innovative, was also constrained by the number and resolution of available images. This limitation potentially affects the accuracy and granularity of the features extracted for obesity rate prediction, particularly in areas where medium-resolution satellite imagery was not available or was of lower quality.

Another significant limitation arises from the DCNN and ResNet-50 features used in our study. While these methods are state of the art in image analysis and feature extraction, their effectiveness is inherently tied to the quantity and quality of the input data. The pretrained ResNet-50 is not fully optimized for the specific nuances of satellite image analysis related to obesity rate prediction. Therefore, this study’s findings must be interpreted with caution, acknowledging that the models used, although advanced, might not capture the complete range of factors influencing obesity rates as discernible from satellite imagery.

### Conclusions

Our study advances the field of spatial regression modeling by integrating DNVFs with traditional models to analyze the geographical distribution of obesity rates across Missouri. This innovative approach, evidenced by high *R*^2^ values, not only underscores the efficacy of incorporating ML in public health analytics but also provides a methodological framework for similar studies.

While our findings contribute significantly to understanding the spatial dynamics of obesity, they are tempered by limitations, such as the reliance on self-reported BMI data, which may underestimate true obesity rates due to reporting biases. Furthermore, the study’s focus on Missouri restricts its broader applicability, suggesting the need for expanded geographic research that includes more diverse populations and environmental settings.

Future research should aim to incorporate longitudinal and multiregional analyses, integrating additional variables, such as socioeconomic status, access to health care, and urbanization levels. These expansions will enable a more comprehensive assessment of the factors influencing obesity and facilitate the development of targeted, effective public health interventions. In addition, we plan to expand our use of RS data by incorporating high-resolution imagery and time-series data in future studies. This approach will enable a more detailed analysis of the dynamic changes in the built environment and their impact on obesity rates, enhancing the precision and applicability of our findings for public health strategies.

By addressing these limitations and exploring these future directions, subsequent research can build upon our findings to enhance the predictive accuracy of obesity prevalence models and ultimately support more nuanced and effective public health strategies.

## Abbreviations

CDC: Centers for Disease Control and Prevention
CNN: convolutional neural network
DCNN: deep convolutional neural network
DL: deep learning
DNVF: deep neural visual feature
ESA: exploratory spatial analysis
FCN: Fully Convolutional Network
GIS: geographic information system
GWR: geographically weighted regression
LISA: local indicators of spatial association
ML: machine learning
PRISMA: Preferred Reporting Items for Systematic Reviews and Meta-Analyses
PSPNet: pyramid scene parsing network
ResNet-50: Residual Network-50
RS: remote sensing
TIGER: topologically integrated geographic encoding and referencing

## Data Availability

The datasets generated and analyzed during this study are not publicly available but are available from the corresponding author on reasonable request.
